# A High Q-Factor Outer-Frame-Anchor Gyroscope Operating at First Resonant Mode

**DOI:** 10.3390/mi11121071

**Published:** 2020-12-01

**Authors:** Bo Jiang, Yan Su, Guowen Liu, Lemin Zhang, Fumin Liu

**Affiliations:** 1 School of Mechanical Engineering, Nanjing University of Science and Technology, Nanjing 210094, China; bjiang@njust.edu.cn; 2 School of Aeronautics and Astronautics, Zhejiang University, Hangzhou 310058, China; gwliusc@126.com; 3 Beijing Institute of Aerospace Control Device, Beijing 100854, China; leminzhang1990@163.com

**Keywords:** MEMS device, gyroscope, outer-frame-anchor, high-quality factor

## Abstract

Disc gyroscope manufactured through microelectromechanical systems (MEMS) fabrication processes becomes one of the most critical solutions for achieving high performance. Some reported novel disc constructions acquire good performance in bias instability, scale factor nonlinearity, etc. However, antivibration characteristics are also important for the devices, especially in engineering applications. For multi-ring structures with central anchors, the out-of-plane motions are in the first few modes, easily excited within the vibration environment. The paper presents a multi-ring gyro with good dynamic characteristics, operating at the first resonant mode. The design helps obtain better static performance and antivibration characteristics with anchor points outside of the multi-ring resonator. According to harmonic experiments, the nearest interference mode is located at 30,311 Hz, whose frequency difference is 72.8% far away from working modes. The structures were fabricated with silicon on insulator (SOI) processes and wafer-level vacuum packaging, where the asymmetry is 780 ppm as the frequency splits. The gyro also obtains a high Q-factor. The measured value at 0.15 Pa was 162 k, which makes the structure have sizeable mechanical sensitivity and low noise.

## 1. Introduction

The mechanical vibratory gyroscope has made significant progress using micromechanical technology. Disc resonator gyros (DRG) [1,2] and quadruple mass gyros [3,4] have been focused on in recent years due to the mode-match operating mechanism [5]. The nonfrequency difference between driving and read-out modes means more massive mechanical gain and sensitivity. DRG works at “wineglass” vibration modes. It owns a similar kinetic theory with Hemispherical Resonator Gyroscope (HRG), developed initially for aircraft navigation. DRG is an in-plane resonator, and it is complemented through silicon-based MEMS fabrications. The advantages meet the requirements of low-cost, small volume, and high performance [6]. 

Many efforts have been taken to achieve a higher quality factor (Q-factor). The DRG developed by Boeing operates at 14 kHz with an 80 k Q factor [7]. UC Davis and Stanford University research groups reported that a multi-ring device with epitaxial silicon has a Q factor of 80 k and a resonant frequency of 250 kHz [8,9]. Cobweb-Like Disk Resonator Gyroscope has a resonant frequency of 18 kHz and 122.5 k Q-factor [10]. It has been proved that high-quality factors and long decay time are obtained by reducing anchor loss [11], thermoelastic damping, and gas damping. Symmetric supporting design is a sufficient principle providing low anchor loss with minimizing anchor strain. Zener model is generally employed to describe thermoelastic energy loss during vibration. The one-dimensional theoretical model is developed further and verified experimentally [12]. The rules mean that if the thermal relaxation rate is comparable to the beam vibration frequency, then maximum energy will be lost as heat and Q will be a minimum [13,14]. 

For low-frequency devices (e.g., less than 100 kHz), Q-factor increases as the natural frequency decreases. The situation is different for high-frequency resonators (e.g., more than 1 MHz), such as the solid disc with nanometer-level gaps [15]. Gas damping is another critical source of energy loss practically. The Q-factor caused by gas damping increases exponentially as the reduction in packaging pressure. The inflection point is around 0.01 Pa, below the value, the gas damping is ignored compared with structural damping [16].

Many works have greatly improved the Q-value, sensitivity, and bias stability [17,18,19,20]. However, a few reports discuss the distribution of vibration frequency characteristics, although it is a fundamental issue. The paper provides a DRG with multi-rings supported through the outer frame. The frequency response distributions are demonstrated through modal analysis and harmonic response analysis. The operating mode (2θ motion) was adjusted as the first mode, far away from the others. Thermoelastic damping analysis and structural optimization for the structure were carried out. SOI processes fabricated the gyroscope with (111) silicon [21], which has two degenerated modes at 2θ mode without frequency splitting theoretically. Open-loop frequency scan experiments addressed the frequency characteristic of the sample. The chip with the average frequency split of 13 Hz was selected and measured in the paper. The bias voltage was also used for mode-match with electrostatic negative stiffness effect. The paper also illustrated the relationship between bias voltage and the resonant frequency of two degenerated modes. The Q-factor was tested by the ring-down method and calculated with fitting free oscillation curves. The quality factors at different ambient pressure reveal that the structural damping dominates when air pressure is below 0.3 Pa for the structure.

## 2. Materials and Methods

### 2.1. Kinetic Equations and Error Analysis

The DRG with multi-rings satisfies the kinetics rules of Coriolis vibratory gyros (CVGs). The motion equations could be specialized to the two-dimensional oscillator. The kinetic equations in open-loop could be expressed as follows with neglected acceleration items.

(1) $$\left[\begin{array}{c} \overset{¨}{x}\\ \overset{¨}{y} \end{array}\right]+\left[\begin{array}{cc} \frac{2}{\tau }+\Delta \frac{1}{\tau }\cos2\theta_{\tau } & -\Delta \frac{1}{\tau }\sin2\theta_{\tau }\\ -\Delta \frac{1}{\tau }\sin2\theta_{\tau } & \frac{2}{\tau }-\Delta \frac{1}{\tau }\cos2\theta_{\tau } \end{array}\right]\left[\begin{array}{c} \dot{x}\\ \dot{y} \end{array}\right]+\left[\begin{array}{cc} \omega^{2}+\omega \Delta \omega \cos2\theta_{\omega } & -\omega \Delta \omega \sin2\theta_{\omega }\\ -\omega \Delta \omega \sin2\theta_{\omega } & \omega^{2}-\omega \Delta \omega \cos2\theta_{\omega } \end{array}\right]\left[\begin{array}{c} x\\ y \end{array}\right]=\left[\begin{array}{c} F_{d}\cos\omega_{d}t\\ F_{y} \end{array}\right]+2k\Omega \left[\begin{array}{c} \dot{y}\\ \dot{x} \end{array}\right]$$

where 1/*τ* is the average value of decay time, and ∆*τ* is the decay time differences along two axes. The character *ω* is the average value of resonant frequency, and ∆*ω* is the difference of natural frequencies at two axes. *θ_τ_* and *θ_ω_* are the deflection angles of the stiffness axis and damping axis with the motion, respectively. *F_d_* and *ω_d_* are the amplitude and frequency of the driven force. The character *k* and *Ω* in the second item on the right represent the Coriolis coupling coefficient and angular velocity.

The first item on the left of the formula is the acceleration term. The second one is the damping matrix. The diagonal elements represent the Q values of the X and Y axes in the coordinate system, and the nondiagonal elements of which are the coupling damping. The stiffness matrix in the third term on the left has the same explanation. The external forces terms are placed at the equation’s right, where *F_y_* equals 0 in the open-loop mode. 

The equation provides access to open-loop output analysis. The B-axis has an orthogonal output applying a driven force along A-axis with the deflected stiffness axis and frequency difference. The output amplitude ratio of the B-axis to A-axis is derived and expressed as

(2) $$\frac{b}{a}=\frac{\omega \Delta \omega \sin2\theta_{\omega }}{\omega^{2}-\omega \Delta \omega \cos2\theta_{\omega }-\omega_{a}{}^{2}}.$$

**Figure 1:**
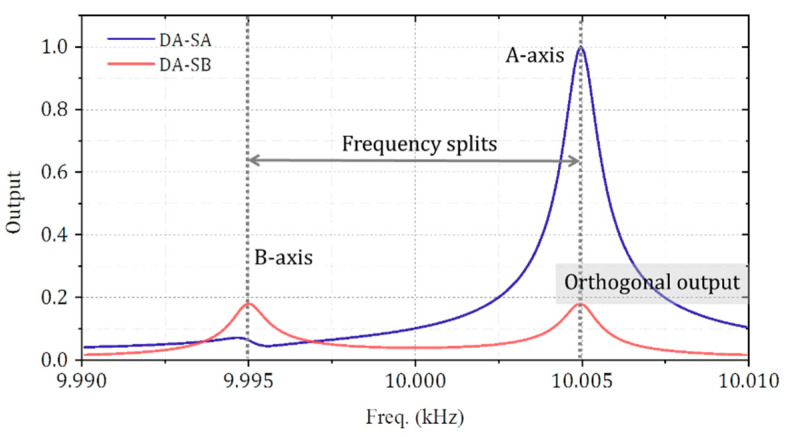
The output relationship of the sensitive axis (A-axis) and orthogonal axis (B-axis) due to the stiffness axis deflection and frequency splits in a two-degree-of-freedom vibration system.

For a two-degree-of-freedom vibration system with the 10 Hz frequency splits, the sensitive axis, and orthogonal axis output with different driven frequency is illustrated in Figure 1. The stiffness axis deflection is 10° in this case. There are two outputs of the B-axis when driven force is applied along A-axis. One is the response at the intrinsic resonant frequency of the B-axis, where the value is 9.995 kHz. The other one is the orthogonal output at the A-axis resonant frequency. The output ratio at 10.005 kHz is 5.68, consistent well with the formula.

### 2.2. The Design of Multi-Rings Structures Operating as the First Mode

The DRG works in degenerated 2θ or 3θ modes without frequency differences typically. For central anchor multi-rings DRG, some in-plane modes (e.g., 1θ) and out-of-plane modes are easily motivated. The out-of-plane resonate modes have low frequency, such as “teeterboard” modal shape, “tub” modal shape, and “saddle” modal shape. For in-plane vibration, resonating in 1θ mode is also easily motivated than 2θ or 3θ modes. 

**Figure 2:**
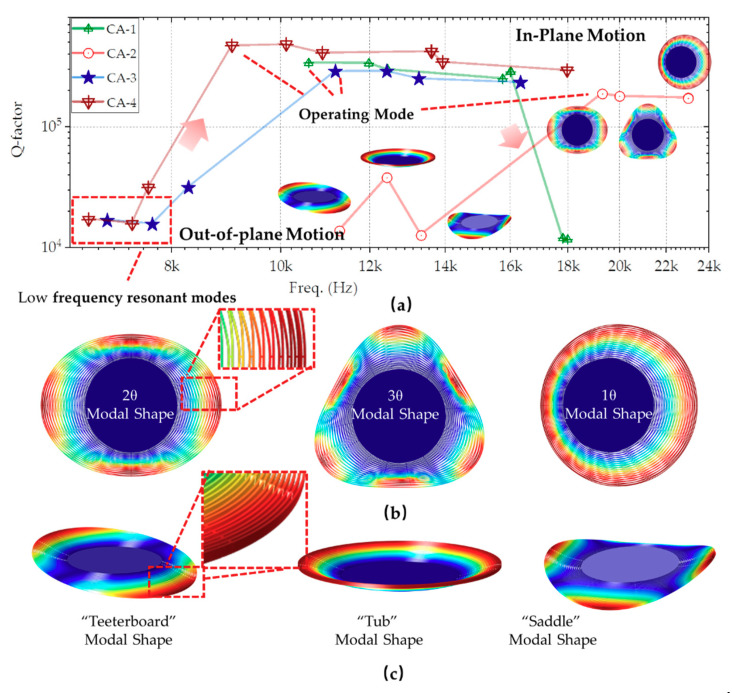
(**a**) Modal shape distributions of multi-rings DRG with a central anchor, (**b**) the in-plane motion modal shapes, and (**c**) the out-of-plane motion modal shapes.

The modal analysis of the central anchor multi-ring structure is listed in Table 1, and the corresponding modal distribution is illustrated in Figure 2. According to the results, the first or even the first few modes are out-of-plane modes. The differences between working motion and others are different with different parameters. The best one is around 10%, generally, where the working mode is easily disturbed by the surrounding modes. The Y-axis in the figure is the Q-factor. When the vibration frequency of the external disturbance is close to the intrinsic modes, even with a small amplitude, the unexpected interference motions are excited due to a high Q-factor. Increasing the resonant frequency is one of the critical solutions to improve the antivibration characteristics.

For some of the high-performance gyros, the first several modal frequencies are generally lower to widen the distance between the working mode and the disturbing mode. It significantly reduces the antivibration ability. On the other hand, higher operating frequencies also mean more significant frequency splits with the same fabrication errors. It is challenging to select a balanced point for better bias instability resisting environmental vibrations disturbance.

The paper adjusts the operating mode as the first mode. It widens the frequency gaps between the operating mode and others to overcome the problem. The structure is supported through an outer frame. The width of the rings is 12 microns, with a thickness of 100 μm. The trenches between rings are 4 microns, meeting the requirement of a 25:1 aspect ratio in silicon deep etching. The structure suppresses the out-of-plane vibration with a compact design, which improves the stiffness in the z-axis direction.

**Figure 3:**
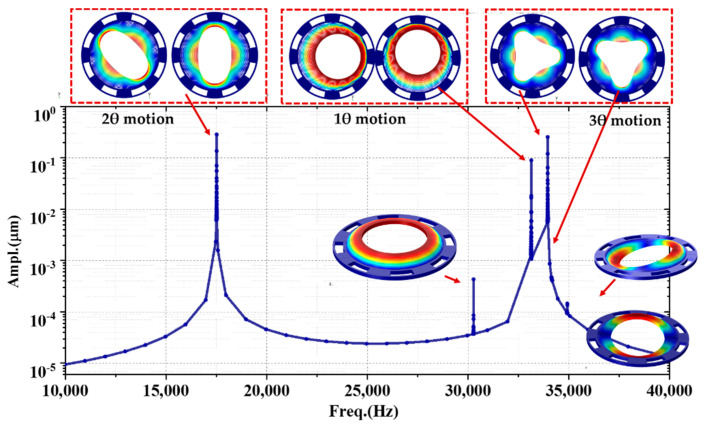
The spectral distributions and motion shapes of the outer-frame supporting multi-rings through harmonic analysis.

The harmonic analysis was carried out, and the frequency spectral is illustrated in Figure 3. The resonate frequency in 2θ modes are 17,540 Hz served as working motion shapes. For (111) silicon, the 2θ modes degenerate without frequency splits identically. The angle between the two orthogonal axes is 45°. The nearest interference mode is located at 30,311 Hz, whose frequency difference is about 72.8% far away from working modes. The mode is an out-of-plane motion, the response amplitude of three orders of magnitude lower than 2θ modes. The nearest in-plane motion is 33,180 Hz, resonating in 1θ modes, which are also degenerated with a 90° angle.

### 2.3. Multi-Rings Structures with Low Thermoelastic Damping

High Q-factor means low energy dissipation, which is one of the critical parameters to judge a gyroscope performance. For multi-rings architecture, the thermoelastic damping plays a critical part in energy dissipation, primarily when the resonator works in high vacuum environments. 

In terms of thermoelastic damping, the maximum energy dissipation occurs when the resonant frequency equals Debye frequency. For the multi-ring structure, the Debye frequency is at megahertz, which means that a higher Q factor is obtained with the lower frequency. 

**Figure 4:**
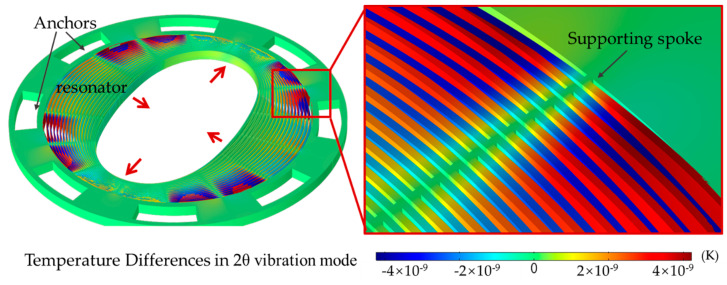
Thermoelastic damping analysis about the outer-frame supporting multi-rings.

The width of the ring determines the distribution of the heat field during vibration, where a narrower ring leads to lower energy consumption. The structural parameters, including the number, width, and radius of the vibration rings, determine the frequency distribution and Q value of the resonator. The results carried out through thermoelastic damping analysis were illustrated in Figure 4 and Table 2. The Q-factor was 0.427 million at 2θ modes. The energy consumption is much larger, oscillating in out-of-plane motion. The quality-factor is 16 k, one order lower than in-plane modes. From the results, the structure described in the paper has higher Q-values and higher first-order modes. Both of them are critical factors to address high-performance gyroscope and good vibration resistance. 

### 2.4. MEMS Fabrications Processes

**Figure 5:**
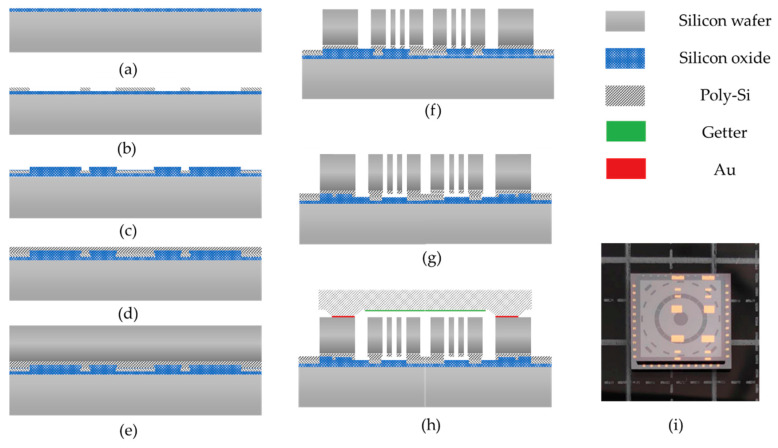
Fabrication process flow of the outer-frame supporting multi-rings: (**a**) thermal oxidation of the substrate silicon wafer inside the oxidizing furnace; (**b**) polysilicon deposition through LPCVD and patterned with RIE; (**c**) oxide deposition as an insulating layer through PECVD; (**d**) polysilicon deposition as anchors; (**e**) silicon–silicon wafer-level bonding and thinning; (**f**) DRIE (deep reactive ion etching), the structure layer; (**g**) release the sacrifice layer through gaseous hydrofluoric acid; (**h**) wafer-level vacuum packaging through Au-Si bonding; and (**i**) gyro die.

Figure 5 reveals the fabrication processes of the outside anchor multi-ring. The bottom layers consist of the substrate wafer, the first oxide layer, the buried layer, and the second oxide layer (from Figure 5a–d). The thermal oxidation was complemented inside the oxidizing furnace. The thickness of the silicon oxide was about 1–2 μm, leading to electrical insulation between the device and the substrate. The ploy-silicon with highly doped boron was deposited through Low-Pressure Chemical Vapor Deposition (LPCVD). The electrical resistivity of the buried layer is about 0.01–0.02 Ω∙cm. The electrical wires and pads were patterned through reactive ion etch (RIE). The second silicon oxide layer was deposited through PECVD at 350 °C as an insulating layer afterward. The silicon oxide was etched, and after that, the second ploy-silicon layer was deposited. 

The device layer was formed through silicon to silicon directly bonding at room temperature. The annealing at 800 °C in a nitrogen atmosphere is also needed for strong covalent bond formation. The device layer is 100 μm, defined by silicon thinning using the CMP (chemical mechanical polishing) process. The multi-rings structure was formed by deep silicon etching (DRIE, deep reactive ion etching) with Bosch Processes. During DRIE, the second oxide layer acts as the etching stopping layer. Then, the sacrifice layer was released through gaseous hydrofluoric acid, preventing adhesion due to liquid surface tension. The device layer is 100 μm and fabricated by (111) silicon wafer, which exhibits isotropy in 2θ motion (from Figure 5e–g).

The wafer-level vacuum package was used afterward (Figure 5h). The cap wafer was fabricated, including cavity etching and getter sputtering. The Au-Si eutectic melt bonding was carried out in a vacuum environment, which is beneficial to improve the quality factor (Q-factor) of the device. 

**Figure 6:**
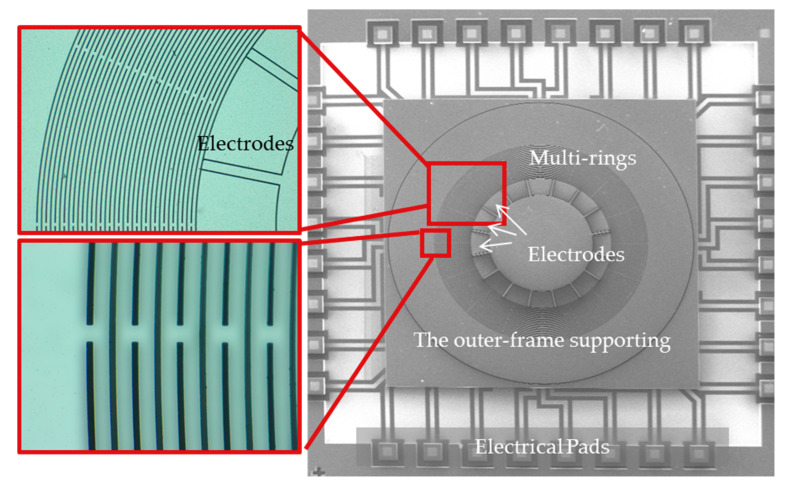
The device layer photography of the outer-frame supporting multi-rings gyroscopes.

The structures after fabrication processes were indicated in Figure 6. There are 16 capacitive electrodes inside the multi-rings. These electrodes have the functions of driving, read-out, orthogonal inhibition, and mode matching. 

## 3. Results

### 3.1. The Frequency Splits and Mode-Matching

**Figure 7:**
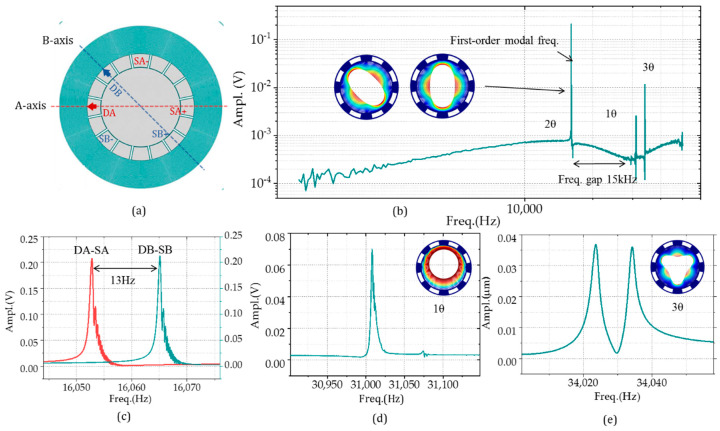
The frequency–amplitude response spectrum of the outer-frame supporting multi-ring gyroscopes: (**a**) the driven and read-out layout of the sweep frequency experiments, (**b**) the amplitude outputs with the sinusoidal signal excitation from 1 to 50 kHz, (**c**) the frequency splits of the gyro, (**d**) the 1θ motion modal shape response at 31,008 Hz, and (**e**) The 3θ motion responses at 34,023 and 34,034 Hz.

The harmonic tests of the gyroscope were conducted for frequency characteristics. In the open-loop mode, the device is equivalent to a resonator with two same resonant frequency degenerate modes. For the multi-ring gyroscope, the angle between the two axes is 45 degree, as illustrated in Figure 7a. The frequency response characteristics of the resonator were obtained by extracting the differential read-out signal with the opposite phase at the detection electrodes (SA+/SB+ and SA-/SB-) by applying different frequency driving forces at the driving electrode (DA and DB). From 1 to 50 kHz, the first-order mode acts as 2θ motion, with the frequency of about 16.6 kHz, which means that the structure is insensitive to any low-frequency signal as shown in Figure 7b–e. This feature enhances the antivibration performance of the gyro. The second-order modal frequency is 31 kHz, far away from the working one, improving motion stability. The 3θ motion mode is located at 34,023 and 34,034 Hz, with the same response amplitude on both axes. The frequency splits could also be obtained through the scanning frequency experiments. Figure 7c addresses two frequency response curves of the A-axis and B-axis, respectively. The frequency differences between two axes after fabrications are about 13 Hz, with 780 ppm frequency differences. 

**Figure 8:**
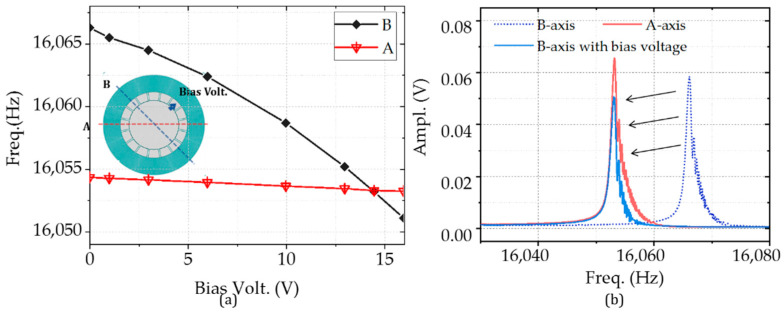
Mode matching experiments with the tuning voltage: (**a**) the relationship between the resonant frequency at 2θ mode and the bias volt applied at the B axis and (**b**) the frequency responses with and without bias at 14.5 V voltage.

The tuning electrodes with variable-gap capacity were employed for mode matching. The electrostatic stiffness is as follows:

(3) $$k_{el}=-\frac{\partial F_{el}}{\partial y}=-\frac{\varepsilon A}{{\left(y_{0}+y\right)}^{3}}V_{DTB}{}^{2}$$

where the *ε* is the dielectric coefficient constant, and *A* is the capacity area, *y*_0_ and *y* are the capacity gaps, *V_DTB_* is the tuning voltage applied on B-axis. Figure 8a indicates that the electrostatic stiffness is the square of bias voltage. There is little change (from 16,054.3 to 16,053.2 Hz) with the tuning voltage from 0 to 16 V for A-axis. In the same condition, the frequency variation on B-axis is 15.2 Hz. The experimental results mean that a voltage of 14.5 V is needed for the mode matching for the gyroscope. The resonant frequency moves to 16,053 Hz. The amplitude is reduced due to the stiffness hardening, as illustrated in Figure 8b. 

**Figure 9:**
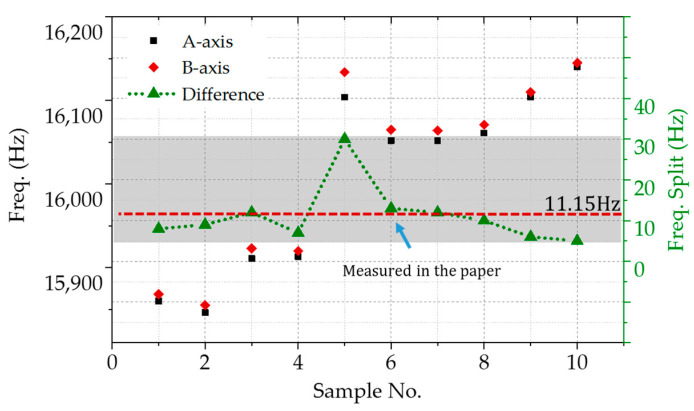
The distribution of two-axis degenerate modes and frequency splits.

By the way, the errors of MEMS fabrication make random distribution of the frequency splitting. Two-axis degenerate modes are illustrated in Figure 9. The resonate frequency ranges from 15,850 to 16,150 Hz, with a span of 300 Hz. The frequency splits are between 5 and 30 Hz, whereas the average value is 11.5 Hz. The No. 6 sample was used and characterized, whose frequency difference is closer to the average.

### 3.2. Q-Factor Characterization and Evaluation

The paper presents the outer-frame supporting multi-ring gyroscopes with 16 kHz operating frequency, optimizing the structure with low energy dissipation. The Q-factor tests were conducted through the ring-down method. The differential signals between SA+ and SA- were read out after the gyroscope was in resonance state through the self-excited closed-loop circuit. The driving-input was grounded so that the resonator energy resonator is in free oscillation. The oscillation amplitude decreased due to the energy dissipation and a high-speed acquisition card collected data. The relationships between ambient pressure and the Q-factor of the structure were also investigated. Laser drilling was used to connect the multi-ring resonator with the atmosphere. Different ambient pressure was obtained by changing the vacuum of the chamber. 

**Figure 10:**
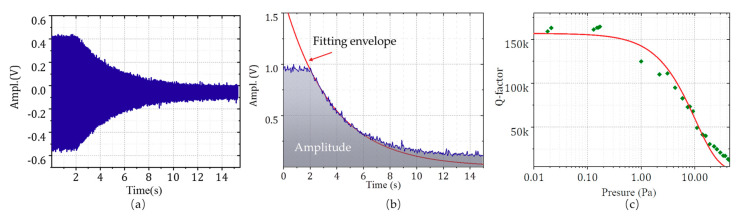
Ring-down Q factor tests: (**a**) the amplitude of the oscillation curve without driving signal; (**b**) The oscillation amplitude envelope, and (**c**) the relationships between ambient pressure and the Q-factor.

Figure 10a,b indicates the attenuation curve at 0.15 Pa and the oscillation amplitude envelope. The variation of oscillation amplitude is derived as the following expression.

(4) $$X=X_{0}e^{-\xi w_{n}t}\sin\left(\sqrt{1-\xi^{2}}w_{n}t+\varphi \right)$$

where decay time constant *τ*_0_ = 1/(*ξ* × *ω_n_*), *ξ* is the damping ratio, and *ω_n_* is the resonant angular frequency and *f*_0_ = *ω_n_*/2π. The Q-factor is expressed as

(5) $$Q=\frac{1}{2\xi }=\pi \tau_{0}f_{0}$$

The Q-factor at 0.15 Pa is 162,237 as of the results. The Q-factor is exponentially related to the ambient pressure, as shown in Figure 10c. The structure is less sensitive to gas damping. Even in the vacuum of 5 Pa, the Q value is also as high as 50,000. The inflection point appears between 0.3 and 1 Pa, where air pressure is no longer the main factor of damping below the point [22]. 

## 4. Discussion

**Figure 11:**
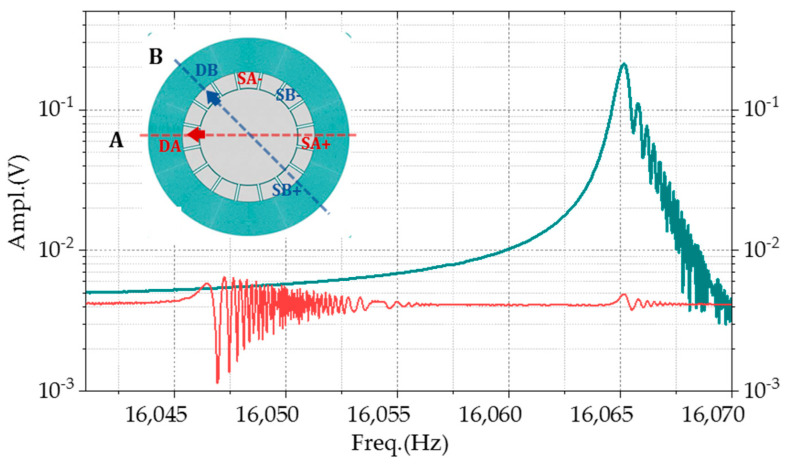
The sensitivity and orthogonal output of B-axis in open-loop. DB-SB means both driving and read-out electrodes were applied at B-axis. DB-SA (the orthogonal signal) means the driving signal is at B-axis and the read-out electrode is located at A-axis.

The gyroscope presented in the paper works in the first mode with a high-quality factor. Compared with the device operating at more than 100 kHz, nanoscale gap capacitance is necessary to provide significantly enough driving and detection signals [15,23]. The minimum gap of the structure is 4 μm, obtained without submicron scale fabrication processes. For this kind of device, the operating frequency is relatively high [24]. The frequency splits between two degenerate axes that were 5 and 30 Hz, and the average value is 11.15 Hz. The sample measured in the paper has 13 Hz frequency splits, which is equivalent to 780 ppm caused by fabrication. The frequency of the A-axis is lower than that of the B-axis, which indicates that there is a systematic error caused by fabrication or packaging. The system error can be compensated by adjusting the spoke angle to realize the structure with a lower frequency difference further [25]. According to Figure 11, the output amplitude ratio of the orthogonal axis to the sensitive axis is 0.0034, and it means the stiffness axis deflection is pretty small. The energy dissipation of the structure is also low. The Q-factor is still 50 k in a 10 Pa environment, which is not difficult to achieve in vacuum packaging. 

## 5. Conclusions

The paper presents a disc gyroscope with an outer-frame anchor multi-rings. The operating mode is the first resonate mode of the structure, improving the antivibration characteristics. The nearest disturbing frequency is 31 kHz, with a 15 kHz difference with operating modes. As per the experimental results, 14.5 V bias voltage was needed for mode-match tunning. The structure optimization for low thermoelastic energy dissipation was also conducted, and 162 k Q-factor was measured at 0.15 Pa. The structure has no low-frequency interference mode, especially the low-frequency out of plane mode, which significantly improves the stability and antivibration characteristics. High-Q design makes the structure to have greater mechanical sensitivity and lower noise. It is a high-performance MEMS gyroscope that can be applied in engineering.

## Figures and Tables

**Table 1:** The structural parameters of central anchor multi-rings.

No.	Maxi. Radius (mm)	Rings Width (μm)	Spoke Width (μm)	The Gap Width between Rings (μm)
CA-1	2.5	12	12	3.5
CA-2	2.5	12	12	7
CA-3	2.5	12	24	3.5
CA-4	2.5	10.5	10.5	3.5

**Table 2:** Q-factor caused by thermoelastic damping of the first six modes.

No.	Frequency (Hz)	Q-Factor	Resonant Modal Shapes
1	17,540	4.2694 × 10^5^	2θ Mode
2	17,540	4.2694 × 10^5^	2θ Mode
3	30,312	1.6208 × 10^4^	“Tub” shape
4	33,180	2.5858 × 10^5^	1θ Mode
5	33,180	2.5843 × 10^5^	1θ Mode
6	33,999	3.6553 × 10^5^	3θ Mode
